# Portal vein pseudoaneurysm secondary to pancreatic lymphoma and biliary stent insertion: a rare cause of haemobilia

**DOI:** 10.1186/s42155-018-0011-7

**Published:** 2018-06-28

**Authors:** Henry Walton, Dominic Yu, Charles Imber, George Webster

**Affiliations:** 1 Royal Free Hospital, Pond Street, Hampstead, London, NW3 2QG UK; 2 University College London Hospital, Euston Road, London, NW1 2BU UK

**Keywords:** Haemobilia, Gastrointestinal bleeding, Portal vein pseudoaneurysm, Transhepatic vascular stent-grafting

## Abstract

**Background:**

Haemobilia, defined as bleeding from the biliary tree, is a rare entity. The most common cause of haemobilia is iatrogenic trauma, which accounts for 70% of cases. Pseudoaneurysms of the portal vein are an extremely rare cause of haemobilia with only four reported cases to date. Conservative treatment, open surgical repair and percutaneous trans hepatic stent-grafting have all been employed in these cases. This displays the lack of consensus regarding the treatment of this condition.

We report the first case of a portal vein pseudoaneurysm following endoscopic common bile duct stent placement performed to relieve obstruction of the common bile duct for lymphomatous infiltration of the pancreatic head. The pseudoaneurysm was successfully treated by placement of a percutaneous trans hepatic covered stent-graft.

**Case presentation:**

A 42-year-old man with a history of lymphomatous infiltration of the pancreatic head and recent endoscopic common bile duct stent placement presented with sudden onset large volume haematemesis. On the portal venous phase of a triple phase CT, this was found to be secondary to a portal vein pseudoaneurysm bulging into the upper portion of the indwelling biliary stent. The pseudoaneurysm was successfully treated by percutaneous trans hepatic placement of a covered vascular stent-graft.

**Conclusions:**

We report a rare case of portal vein pseudo aneurysm successfully treated by percutaneous trans hepatic portal venous covered vascular stent-graft insertion.

## Background

Haemobilia, defined as bleeding from the biliary tree, is a rare entity that was first described in 1948 by Sandblom et al. (1948). The most common cause of haemobilia is iatrogenic trauma, which accounts for 70% of cases. Accidental trauma now only accounts for 5% of cases. Important causes of non-traumatic haemobilia include rupture of hepatic artery aneurysms, hepatic abscesses, cholangitis, hepatobiliary tumours, hepatic cysts and coagulopathy (Zaydfudim et al., 2014). Pseudoaneurysms of the portal vein are an extremely rare cause of haemobilia with only four reported cases (Javadrasshid et al., 2012; Wallis et al., 2010; Weber et al., 2016; Ierardi et al., 2016).

We report the first case of a portal vein pseudoaneursysm following endoscopic common bile duct (CBD) stenting performed to relieve obstruction of the CBD for lymphomatous infiltration of the pancreatic head.

### Incidence

Life-threatening haemobilia is rare but carries a high mortality, quoted as 25% in large retrospective studies (Zaydfudim et al., 2014). The majority of cases of symptomatic haemobilia result from a hepatic artery-to-biliary tree fistula (Chin & Enns, 2010). In contrast, symptomatic haemobilia from a portal vein or hepatic venous abnormality is extremely rare due to the relatively low pressure gradient between these systems. There have only been four reported cases of symptomatic haemobilia from a portal vein pseudoaneurysm. The four previously reported cases are summarized in Table 1.

**Table 1:** Published cases of portal vein pseudoaneurysm

Author	Patient	Cause	Treatment
Walis et al., (2010)	10 years, Male	Blunt abdominal trauma falling from a bicycle	Conservative management
Javadrasshid et al., (2012)	28 years, female; 32 weeks pregnant	Spontaneous	Left hepatectomy
Weber et al., (2016)	69 years, female	Seatbelt injury during a road traffic accident	Percutaneous portal vein stent
Ierardi et al., (2016)	42 years, male	Blunt abdominal trauma during a road traffic accident	Percutaneous portal vein stent

Summary of reported cases of portal vein pseudoaneurysms

### Presentation

Patients with haemobilia classically presents with ‘Quincke’s triad’ of upper abdominal pain, gastrointestinal haemorrhage and jaundice. In practice, the full triad is only present in 22–35% of patients (Navuluri, 2016). When there is slow intermittent bleeding, additional features include melaena and iron-deficiency anaemia.

### Investigation

When haemobilia is suspected, its investigation and treatment requires a multidisciplinary approach involving clinical assessment and a combination of endoscopy, ultrasound, Computed Tomography (CT), Magnetic Resonance Imaging (MRI) or Digital Subtraction Angiography (DSA). The aims of haemobilia management are to identify the site of bleeding, stop the bleeding and prevent secondary biliary obstruction.

### Endoscopy

Positive endoscopic findings in haemobilia include bleeding from the ampulla and blood products within the duodenum. If Endoscopic Retrograde Cholangiopancreatography (ERCP) is performed, a filling defect may be seen within the CBD representing ductal thrombus. When present, the endoscopic findings of haemobilia are clearly highly specific. However, a small series assessing patients with haemobilia revealed direct or indirect evidence of Haemobilia on endoscopy in only 60% of these patients (Navuluri, 2016).

### Ultrasound

Ultrasonography is useful for identifying haemobilia and potential underlying aetiologies. Haemobilia forms avascular tubular lesions of hyper reflectivity filling and expanding the biliary ducts and minimally mobile, hyper-reflective, avascular masses within the gallbladder. With these finding, serial ultrasound showing decreasing reflectivity over time is highly suggestive of haemobilia.

Ultrasound may also identify the underlying cause for haemobilia. In particular, vascular aneurysms and pseudo aneurysms are seen as well circumscribed anechoic masses with internal vascular flow seen on doppler ultrasound. Ultrasound may also be performed at the bedside which is of particular benefit in unstable patients.

### CT angiography

In cases with a high suspicion of haemobilia or confirmed haemobilia on endoscopy, three or four phase CT can be performed. Though no studies have yet attempted to calculate the sensitivity of multiphase CT for identifying haemobilia, intermittent bleeding and slow flow bleeding are thought to cause a high rate of false negative results. However, multiphase CT can identify a specific active bleeding point and display indirect features of haemobilia such as pseudoaneurysms, biliary tree thrombus, biliary dilatation, vascular malformations, hepatic tumours and gallstones.

### MRI

MRI can be used to identify thrombus within the biliary tree, haemorrhagic bile and active haemobilia. Biliary tree thrombus appears as a filling defect on MRI fluid sensitive sequences. Hamorrhagic bile has a high signal intensity on fat-supressed T1-weighted sequences and a low signal on T2 weighted sequences. This may result in a fluid-fluid levels within the bile ducts on heavily T2 weight sequences, with hemorrhagic bile forming the low signal, gravity dependant layer and non hemorrhagic bile forming the high signal upper layer. Active bleeding may be identified if dynamic, contrast enhanced MRI sequences are performed.

### Digital subtraction angiography

DSA is the gold standard for the investigation of haemobilia but is invasive and is usually reserved for when there is an intent to treat suspected haemobilia with vascular embolization. In the context of clinically significant haemobilia, 88–100% of patients will have a bleeding point, pseudoaneurysm or arteriovenous malformation identified on DSA (Zaydfudim et al., 2014; Fidelman et al., 2008; Bloechle et al., 1994).

### Treatment of portal vein pseudoaneurysm

Due to the rarity of symptomatic portal vein pseudoaneurysms, there are no guidelines on their treatment. The four previously published case reports of symptomatic portal vein pseudoaneurysms are summarized in Table 1. The cases exhibit a range of treatment options from conservative management to open surgery.

Wallis et al. described the successful conservative management of a 10-year-old male with a traumatic portal vein pseudoaneurysm following abdominal trauma sustained when falling from a bicycle. The authors argue that the low-pressure of the portal venous system and tamponade effect of the surrounding liver allowed effective haemostasis without complication (Wallis et al., 2010).

Open surgery for portal vein injuries is notoriously high risk with mortality ranging from 50 to 70% in the published literature (Fraga et al., 2009; Pearl et al., 2004). Javadrasshid et al. described a case of severe haemobilia from a left portal vein pseudoaneurysm in a 28-year-old female at 32 weeks of pregnancy. Although endovascular treatment was considered in this case, when the patient became severely hypovolaemic with rapidly worsening haematemesis, the decision was made to perform a life saving left hepatectomy. The patient later delivered by caesarian section and made a full recovery, leaving hospital 14 days after her operation (Javadrasshid et al., 2012).

There have been two cases of percutaneous trans hepatic portal venous stenting for portal vein pseudoaneurysms. The short term outcomes reported in these two published cases have shown rapid cessation of bleeding with no complications following the procedure (Weber et al., 2016; Ierardi et al., 2016).

## Case presentation

**Fig. 1:**
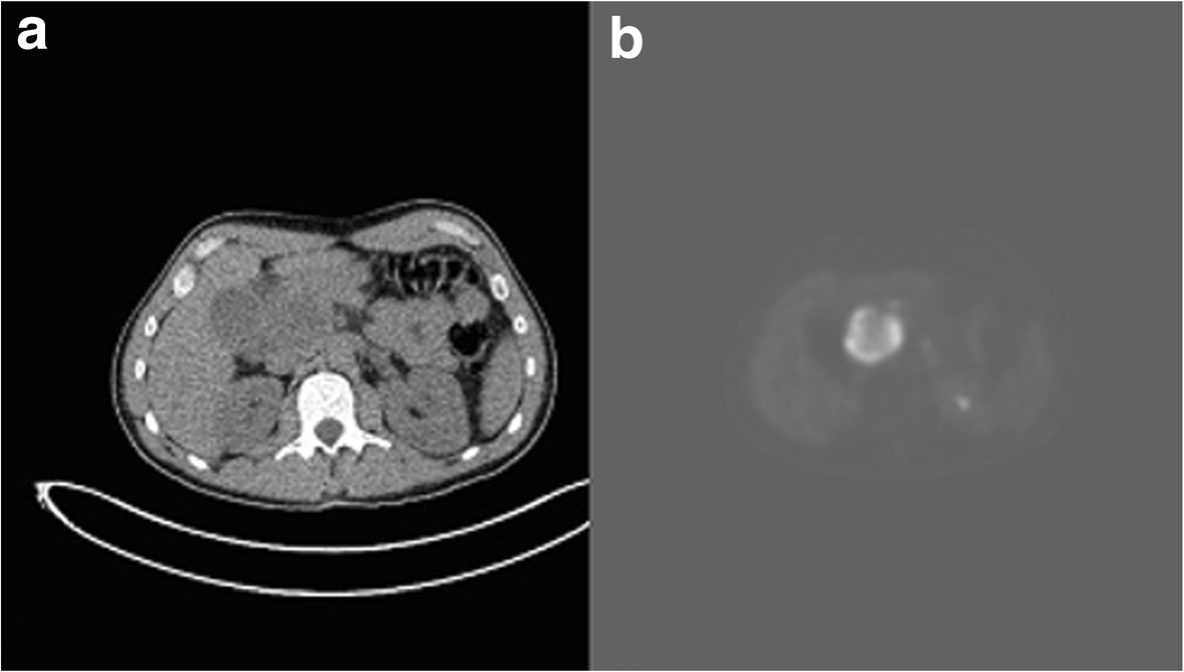
**a** Abdominal CT showing a large periportal nodal mass and **b** Abdominal PET-CT showing avid tracer uptake

A 42-year-old man initially presented to hospital with painless jaundice and weight loss of 9 kg over a period of four to six weeks. A CT scan of the abdomen and pelvis in the portal-venous phase revealed a 5.3 × 4.7 cm periportal lymph node adjacent to the pancreatic head (Fig. 1 a). A Positron Emission Tomography –CT (PET-CT) showed avid tracer uptake (Fig. 1b). There was associated biliary obstruction at the level of the porta hepatis. The patient underwent percutaneous biopsy followed by ERCP and deployment of a 10 mm × 80 mm covered metal stent (WallFlex Biliary RX Stent, Boston Scientific, Marlborough, MA, United States).

The core biopsy of the nodal mass revealed diffuse large B-cell lymphoma (DLBCL). Three weeks following the first cycle of Rituximab, Cyclophosphamide, Doxorubicin, Vincristine and Prednisolone (R-CHOP) chemotherapy, the patient presented to hospital complaining of fever and malaise. The patient had pyrexia of unknown origin and was prescribed a course of Ceftazidime and Metronidazole intravenous antibiotics.

**Fig. 2:**
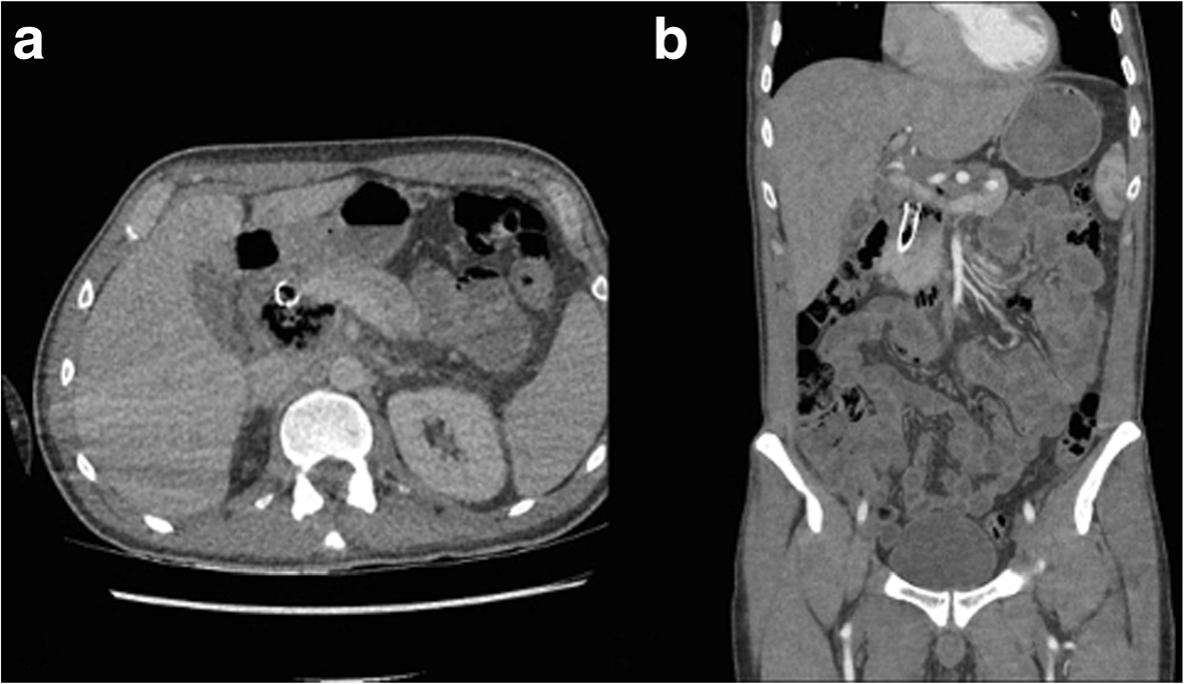
Abdominal CT following one dose of chemotherapy showing necrosis of the periportal nodal mass and erosion of the stent through the bile duct

The following day the patient developed sudden onset haematemesis, passing 500 ml of fresh red blood. An urgent portal venous phase CT of the abdomen showed extensive necrosis of the mass with erosion of the metal biliary stent through the bile duct wall causing a large amount of intralesional gas (Fig. 2).

This was followed promptly by an urgent duodenoscopy that revealed acute blood actively oozing from within the metal biliary stent and fresh haematoma in the second part of the duodenum.

**Fig. 3:**
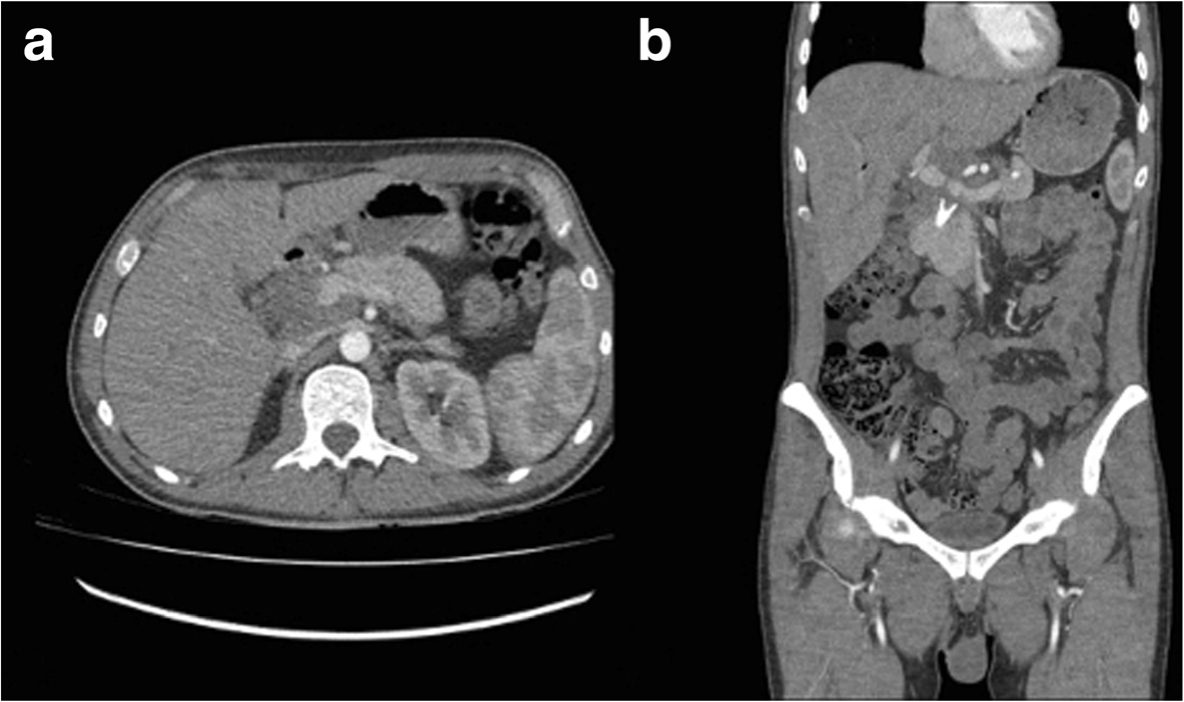
Portal venous phase CT showing a main portal vein pseudoaneurysm closely related to the upper end of the biliary stent

The patient was transferred immediately back to radiology to have a triple phase (pre-contrast, arterial and portal venous phase) CT of the abdomen to assess for a bleeding point. The multiphase CT revealed a 13 mm pseudoaneurysm of the main portal vein. The pseudoaneursym was bulging towards the proximal end of the indwelling biliary stent (Fig. 3). There was also hyperdense material within the stent lumen, which was presumed to be acute blood.

The portal vein pseudo aneurysm was thought to be due to iatrogenic trauma of the portal vein wall at the time of stent insertion with further erosion of the vessel wall in the weeks following insertion. The other possible causes considered were vascular injury at the time of percutaneous biopsy and infection/inflammation within the surrounding lymphomatous mass causing direct vascular erosion. After multidisciplinary team discussion between Interventional Radiology, Hepatobiliary Surgery and Gastroenterology, the decision was made to perform percutaneous transhepatic covered stenting of the main portal vein.

**Fig. 4:**
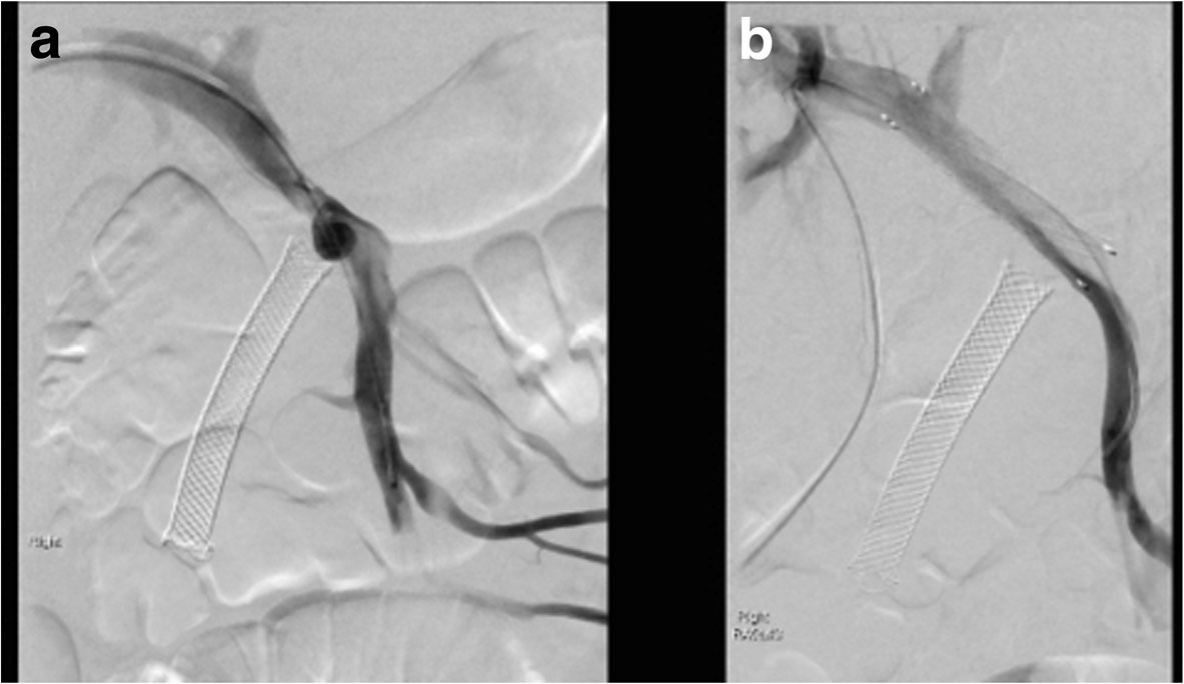
Direct angiography of the pseudoaneursym pre and post-stenting. **a** Digital subtraction venogram showing a large portal vein pseudoaneurysm bulging into the proximal end of the common bile duct stent. **b** Post stent deployment venogram showing immediate exclusion of the portal vein pseudoaneurysm

**Fig. 5:**
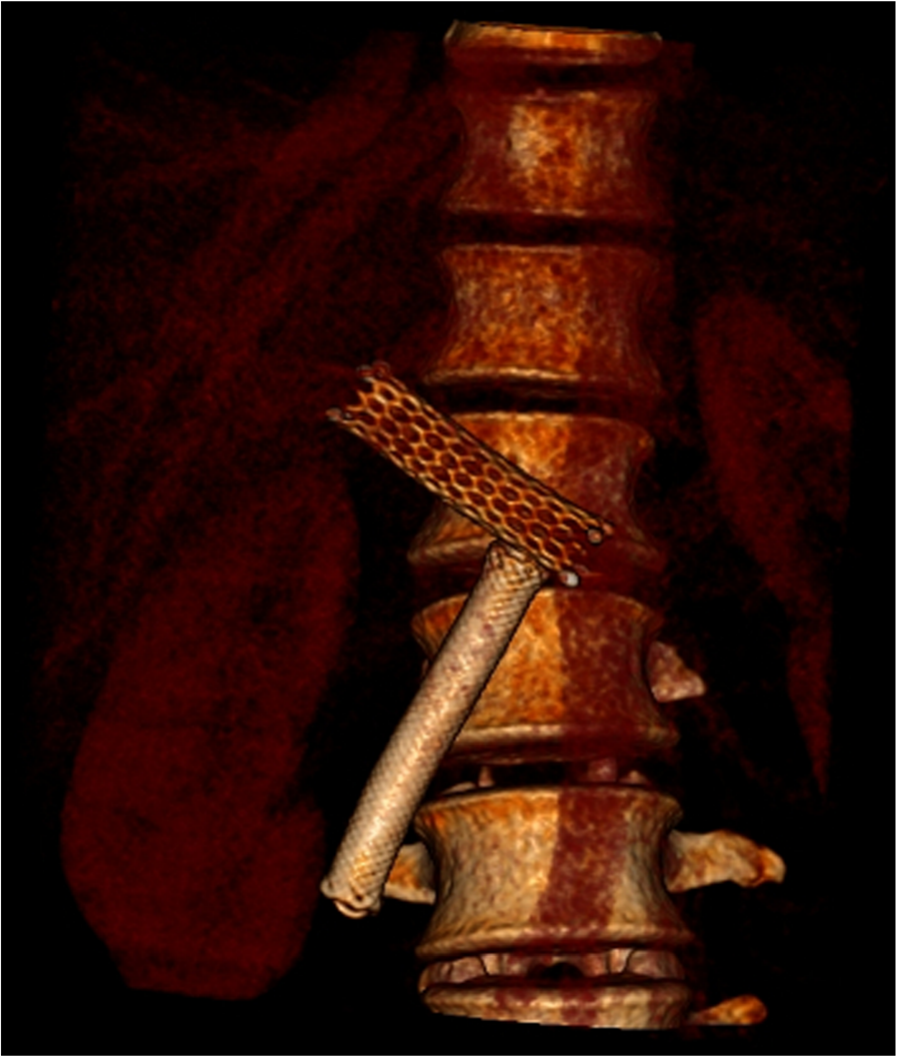
3d CT reconstruction of the covered vascular stent and the biliary stent. 3d reconstruction of a follow-up CT of the abdomen showing the relationship of the portal vein stent and the common bile duct stent. The covered portal vein stent has successfully excluded the pseudoaneurysm

A right anterior portal vein puncture was performed using a 21G micropuncture needle under ultrasound guidance. A 10 Fr sheath was inserted. A C-2 Cobra catheter was positioned in the superior mesenteric vein. Digital subtraction venograms performed via the catheter confirmed a broad-based saccular psudoaneurysm of the main portal vein measuring approximately 13 mm in diameter. The pseudoaneurysm was positioned at the anteroinferior edge of the proximal portal vein and was bulging into the proximal end of the biliary stent (Figs. 4a and 5). There was no active bleeding at the time of the procedure. An Amplatz SuperStiff guidewire was passed to the SMV. A covered 14 mm × 60 mm FLUENCY plus Vascular Stent Graft (Bard Peripheral Vascular, Tempe, AZ, United States) was deployed in the main portal vein across the pseudoaneurysm. There was immediate exclusion of the pseudoaneurysm on post deployment venograms (Fig. 4b). The main portal vein, right portal vein, left portal vein, superior mesenteric vein and splenic vein remained patent. The sheath was removed and the tract plugged with Avitene Microfibrillar Collagen Hemostat (Bard Davol, Warwick, RI, United States). The patient’s haematemesis ceased promptly following the procedure. Post-procedure anticoagulation and antiplatelet treatment was not initiated given the patients risk of further bleeding.

There have been no procedure related complications in over 1 year since the stent placement. The patient continued R-CHOP chemotherapy for DLBCL and was clinically well at recent outpatient follow up.

## Conclusion

Haemobilia secondary to portal vein pseudoaneurysm is extremely. We report a rare case of portal vein pseudo aneurysm successfully treated by percutaneous trans hepatic portal venous covered vascular stent-graft insertion.

## Abbreviations

CBD: Common bile duct
CT: Computed Tomography
DLBCL: Diffuse large B-cell lymphoma
DSA: Digital Subtraction Angiography
ERCP: Endoscopic Retrograde Cholangiopancreatography
MRI: Magnetic Resonance Imaging
PET-CT: Positron Emission Tomography – CT (PET-CT)
R-CHOP: Rituximab, Cyclophosphamide, Doxorubicin, Vincristine and Prednisolone

## Authors’ information

Henry Walton – Interventional Radiology Fellow, Royal Free Hospital, Pond Street, Hampstead, London, NW3 2QG, United Kingdom.

Dominic Yu – Consultant Interventional Radiologist, Royal Free Hospital, Pond Street, Hampstead, London, NW3 2QG, United Kingdom.

Charles Imber – Consultant Surgeon, Royal Free Hospital, Pond Street, Hampstead, London, NW3 2QG, United Kingdom.

George Webster – Consultant Gastroenterologist, University College London Hospital, Euston Road, London, NW1 2BU.
